# No association between binge eating disorder and severity of non‐alcoholic fatty liver disease in severely obese patients

**DOI:** 10.1002/jgh3.12309

**Published:** 2020-03-01

**Authors:** Clémence M Canivet, Pascal Perney, Faredj Cherick, Magalie Orlowski, Stéphanie Patouraux, Béatrice Bailly‐Maitre, Albert Tran, Antonio Iannelli, Philippe Gual, Rodolphe Anty

**Affiliations:** ^1^ Cote d'Azur University, Nice Hospital, INSERM, U1065, C3M Nice France; ^2^ Addiction Medicine Hospital Grau‐du‐Roi Nîmes France; ^3^ Paris‐Saclay University, Paris‐Sud University, UVSQ, CESP, U1018 Villejuif France; ^4^ Cote d'Azur University, Nice Hospital Nice France; ^5^ Cote d'Azur University, INSERM, C3M Nice France

**Keywords:** anxiety, binge eating disorder, bulimia, depression, fatigue, non‐alcoholic fatty liver disease, severe obesity

## Abstract

**Background and Aim:**

The main aim of this study was to evaluate if the binge eating disorders (BEDs) related to obesity were associated with the severity of non‐alcoholic fatty liver disease (NAFLD).

**Methods:**

Severely obese patients who had been referred for bariatric surgery were included in this study at the Nice University Hospital. All patients underwent a liver biopsy at the time of surgery. Between 2008 and 2015, 388 patients had an assessable Bulimia Test (BULIT) self‐questionnaire at the time of surgery. A subgroup (*n* = 183), between 2011 and 2015, also responded to a Beck Depression Inventory, Hospital Anxiety and Depression Scale, and a Fatigue Impact Scale autoquestionnaire. A control group of 29 healthy people matched by age and gender was included.

**Results:**

Among the 388 obese patients (median age 40 years, body mass index 41.7 kg/m^2^, 81% women), 14 patients had a “probable diagnosis” of BED, and 47 patients had a “high risk” of developing a BED according to the BULIT. Obese patients had significantly more severe BED, depression, anxiety, and fatigue compared to controls. Steatosis, non‐alcoholic steatohepatitis, or fibrosis was not associated with BED. Similarly, the severity of NAFLD was not associated with depression, anxiety, or fatigue.

**Conclusions:**

Severely obese patients had more severe BED, depression, anxiety, and fatigue than lean subjects independent of the severity of NAFLD.

## Graphical abstract

Non‐alcoholic fatty liver disease, the emerging liver disease of the 21st century, is related to obesity. However, binge eating disorders, and also frequently associated psychiatric disorders (depression, anxiety, and fatigue), are not associated with the severity of liver pathology in severely obese patients.

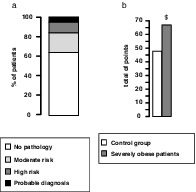

## Introduction

Obesity is a major public health problem. In 2013, the global prevalence of overweight and obese individuals was 36.9% for men and 38% for women,^1^ and the mean body mass index (BMI) increased from 21.7 kg/m^2^ in 1975 to 24.2 kg/m^2^ in 2014 in men.^2^ In Europe and France, the prevalence of obesity is 15.9 and 23.9%, respectively.^3^ In France, morbid obesity (BMI ≥ 40 kg/m^2^) applies to 1% of obese people.^4^, ^5^

Severe and morbid obesity are associated with the development of non‐alcoholic fatty liver disease (NAFLD).^6^ Histological assessment of the liver in cohorts of severely and morbidly obese patients who underwent bariatric surgery showed that 86% exhibited steatosis, 27.7% NAFLD activity score (NAS) ≥ 3, and 0.6% cirrhosis.^7^, ^8^ The heterogeneity of liver damage in patients with severe obesity could be explained by environmental risk factors other than obesity. First, the quality of habitual diet plays a role in the pathogenesis of NAFLD, and both risky (such as fructose) and protective foods (such as those that are part of the Mediterranean diet) have been described.^9^, ^10^ Secondly, NAFLD is also associated with a lack of physical activity and an excessively sedentary lifestyle.^9^ Third, psychological factors can impact both dietetic habits and the overall way of life. For example, junk food and low‐cost food tends to be salty, greasy, and/or sweet and thus highly palatable, particularly if paired with large amounts of sweetened beverages. These foods are highly addictive, and the transition from normal “liking” and “wanting” to addictive behavior is a key neural mechanism in food disorder.^11^ As a matter of fact, the prevalence of binge eating disorder (BED) was higher in NAFLD patients that in the general population in a pilot study,^12^ and BED was found to confer an increased risk of metabolic syndrome.^13^ Moreover, BED co‐occurred with a plethora of psychiatric disorders (71%), most commonly mood and anxiety disorders (53%), in a large cohort of patients presenting to specialist eating disorder clinics in Sweden.^14^, ^15^ However, the association between BED and the severity of the histological features in NAFLD is unknown. We hypothesized that patients with BED and/or psychological factors would have more severe histological NAFLD features than patients without a psychological disorder. To answer this question, we used our cohort of well‐characterized (liver biopsy and biological data) severely obese patients who had been referred for bariatric surgery. The main objective of this study was to assess the severity of BED with respect to liver histology in severely obese patients.

## Patients and methods

### *Severely obese patients*

Between September 2008 and December 2015, 388 severely obese patients referred for bariatric surgery in our hospital completed one self‐report to screen for BED. Patients with overt clinical bulimia nervosa, diagnosed by two specialist psychiatrists (Magalie Orlowski and Faredj Cherick), were contraindicated for surgery as recommended by French guidelines and were not included in our cohort.^16^ Our cohort has been partially described previously.^17^ The study protocol was performed according to French legislation regarding ethics and human research and was approved by the local ethics committee (Huriet‐Serusclat law, DGS 2003/0395).

#### *Preoperative assessment*

Clinicobiological preoperative assessment was performed 2 or 3 weeks before surgery. A systematic review of all the prescribed treatments in the last 6 months was conducted, particularly for anxiolytics, antidepressant therapy, and sleeping pills. No information about job status and education level was obtained from patients. Metabolic syndrome was defined according to Alberti *et al*.^18^ Hepatic wedges were obtained during bariatric surgery by a surgeon specialized in liver surgery (Antonio Iannelli) and were reviewed by a liver pathologist (Stéphanie Patouraux) without knowledge of the clinical or biological characteristics of the patients. The diagnosis was based, for all biopsies, on the Steatosis, Activity, Fibrosis (SAF) score for diagnosis of non‐alcoholic steatohepatitis (NASH) in morbidly obese patients. Liver fibrosis was classified into seven stages according to the NASH Clinical Research Network Scoring System Definition and Scores (more details in supplementary data).

#### *Self‐reports*

Severely obese patients completed self‐screening questionnaires for BED, depression, anxiety, and fatigue 2–3 weeks prior to surgery, and an exploratory group completed these same questionnaires again 1 year after surgery. The revised Bulimia Test (BULIT) is a validated self‐report for the screening of BEDs, translated to French.^19^ The score, based on the sum of 32 of 36 items, ranges from 32 (no symptoms) to 160 (elevated symptoms). A score ≥104 means a “probable diagnosed bulimia nervosa,” a score between 88 and 103 a “high risk of eating disorders,” a score between 73 and 87 a “moderate risk of eating disorders,” and a score <73 indicates no pathological state.^20^, ^21^, ^22^

From 1 July 2011, three other self‐reports were added to evaluate depression (Beck Depression Inventory [BECK], Hospital Anxiety and Depression Scale [HAD]), anxiety [HAD], and fatigue (Fatigue Impact Scale [FIS]). The BECK is a 13‐item self‐report used for the measurement of deep depression. A score of 0–4 means no depression, 5–7 “mild depression,” 8–15 “moderate depression,” and >15 “severe depression.”^23^, ^24^ HAD assesses depression and anxiety through seven items each. A score of 0–7 indicates no pathological state, a score between 8 and 10 is “possible diagnosis,” and a score > 10 is “definite diagnosis” of depression/anxiety.^25^ These tests, in French, have been validated.^26^, ^27^ “Significant” depression was considered if the scores were as follows: BECK >8 or HAD‐depression >10.

The severity of fatigue was measured using the French version of the FIS.^28^ FIS is a self‐report consisting of 40 statements that describe manifestations of fatigue. Each item is rated on a 5‐point scale with a maximum of 160 points.

### *Control group*

Healthy persons working in the scientific institute of our center, matched for age and gender to the patients, without a background of psychological disorders, and without anti‐depressive or anxiolytic drug use, filled out the four self‐reports in July 2016. Written informed consent was obtained from all subjects.

### *Statistics*

Quantitative variables are presented as the median and 25–75th interquartile range. Quantitative values were compared using the Mann–Whitney test or the Student's *t*‐test as required. The Chi‐square test was used to compare qualitative values. All calculations were made using NCSS 2012 (Saugus, MA, USA).

## Results

### *Characteristics of the studied population*

A total of 388 severely obese patients (40 [30–50] years old, BMI = 41.8 [39.4–44.7] kg/m^2^, female gender 81%) were included in our study. Patients with symptomatic BED were contraindicated by a psychiatrist and represented 4% of all the patients (*n* = 1735) referred for bariatric surgery at our hospital during the period between September 2008 and December 2015. Among the patients included in our study, 196 patients (51%) had metabolic syndrome, and 64 (16%) had type 2 diabetes mellitus. Among the 388 patients, 338 (87%) patients had NAFLD, and 71 (18%) had NASH. Significant fibrosis was present in 28% of patients (Table 1). The distribution of liver features was the same as that of another severely obese cohort in Europe.^29^ Only a few patients took antidepressive (7.8%), anxiolytic (5.9%), or sedative drugs (2.6%).

**Table 1:** Characteristics of the population

Variables	Severely obese patients	Control subjects	*P*
Total number of subjects	388	29	—
Female (%)	313 (81)	25 (86)	NS
Age (years)	40 [30–50]	39 [28–44.5]	NS
BMI (kg/m^2^)	41.7 [39.4–44.5]	21.3 [19.7–22.3]	<0.000001
NAFLD (%)	338 (87)		—
NASH (%)	71 (18)		—
Fibrosis ≥ F2 according to Brunt (%)	109 (28)		—
Grade of steatosis (%)			—
S0	50 (13)		
S1	129 (33)		
S2	113 (29)		
S3	96 (25)		
Grade of fibrosis (%)			—
F0	69 (18)		
F1	210 (54)		
F2	94 (24)		
F3	12 (3)		
F4	3 (1)		
Type 2 diabetes (%)	64 (16)	0	<0.000001
HOMA‐IR	4.3 [3.0–7.0]		—
Tobacco use (%)	88 (23)	6	NS
Metabolic syndrome (%)	196 (51)	0	<0.000001

Data are expressed as medians with interquartile ranges and compared using the Mann–Whitney test for quantitative values and or Chi‐square test for qualitative values.

—, Not evaluable; BMI, body mass index; HOMA‐IR, Homeostasis Model Accessment of insuline resistance; NAFLD, non‐alcoholic fatty liver disease; NASH, non‐alcoholic steatohepatitis; NS, not significant.

Among the 388 severely obese patients, 183 filled out three more self‐reports to evaluate depression (BECK + HAD‐Depression), anxiety (HAD‐anxiety), and fatigue (FIS). They were comparable in terms of BULIT and NASH but were older (*P* = 0.047), and they had more men (*P* = 0.02) and less steatosis (*P* = 0.0003) than the first 205 patients who filled out only the BULIT.

The control group, consisting by 29 healthy lean subjects (39 [28–44.5] years old, BMI = 21.3 [19.7–22.3] kg/m^2^, female gender 86%), filled out the same self‐reports. They had no background of depression or psychotropic intake. Lean subjects were comparable to the 388 severely obese patients with respect to gender and age.

An exploratory follow‐up group of 15 patients was recruited to fill out the same four self‐reports 12 months after bariatric surgery. These patients were slightly older (48 *vs* 40 years old, *P* = 0.0043) but were comparable to the whole cohort in terms of gender and BMI at baseline (Table S1, Supporting information).

### *Results of BED self‐report*

**Figure 1:**
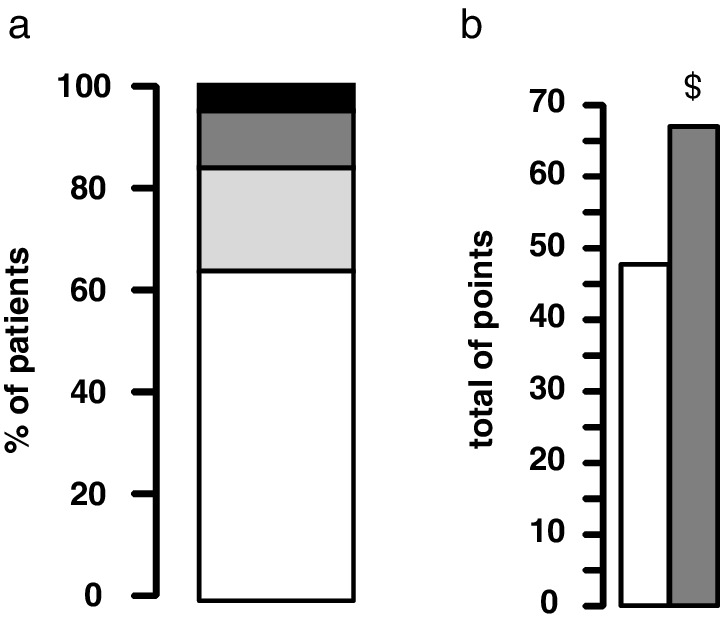
Evaluation of eating disorder through Bulimia Test (BULIT). (a) Distribution of the BULIT values in severely obese patients. No pathology = BULIT <73; moderate risk = BULIT 73–87; high risk = BULIT 88–103; probable diagnosis = BULIT ≥104. (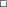), No pathology; (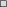), moderate risk; (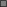), high risk; (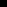), probable diagnosis. (b) Medians of BULIT in severely obese patients and in the control group. ^$^ *P* ≤ 0.00001 *versus* control group. (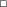), Control group; (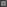), severely obese patients.

In severely obese patients, 14 (4%) patients had a “probable diagnosis” (BULIT ≥104) of bulimia nervosa, 47 (12%) had a “high risk” (88 ≤ BULIT ≤ 103) of eating disorders, 85 (22%) had a “moderate risk” (73 ≤ BULIT <88) of eating disorders, and 242 (62%) had no pathological test (Fig. 1a). Median BULIT score was 66 [56–79] (Fig. 1b). The prevalence of eating disorders (BULIT ≥ 73) was 38%. No pathological self‐report (BULIT ≥ 73) was found in the control group (*P* < 0.00001 *vs* patients), and median BULIT score for the control group was 47 [43–57.5] (Fig. 1b). One year after surgery, the BULIT scores were significantly lower compared to baseline. No difference was found for the prevalence of eating disorders regardless of the presence of NAFLD, NASH, or fibrosis F0–F1 *versus* F2–F4 or fibrosis F0–F2 *versus* F3‐F4 in severely obese patients (Table 2). Likewise, no correlation was found between grades of steatosis, stages of fibrosis, or NAS score and the BULIT. Patients with moderate, high risk, and probable diagnosis of BED (BULIT ≥ 73) had the same histological features as patients with no pathological test (BULIT < 73). Likewise, patients with high risk and probable diagnosis of BED (BULIT ≥ 88) had the same histological features as patients with moderate risk and no pathological test (BULIT < 88). Similarly, patients with a more severe BED (BULIT ≥ 104) had the same histological features as other patients.

**Table 2:** Results of BULIT according to liver lesions, type 2 diabetes mellitus, or metabolic syndrome in 388 obese patients

	Yes/no (*n*)	BULIT	*P*
NAFLD	Yes (338)	66.5 [56–79]	NS
	No (50)	65.5 [57–77]	
NASH	Yes (71)	66 [55–76]	NS
	No (317)	66 [57–80]	
Fibrosis ≥ 2	Yes (109)	66 [56–78]	NS
	No (279)	66 [57–79]	
Fibrosis ≥ 3	Yes (15)	64 [55–83]	NS
	No (373)	66 [57–79]	
Type 2 diabetes	Yes (64)	64 [56.3–75.8]	NS
	No (324)	67 [56–79.8]	
Metabolic syndrome	Yes (196)	68 [57–79]	NS
	No (192)	65 [56–79]	

Data are expressed as medians with interquartile ranges and compared using the Mann–Whitney test.

BULIT, Bulimia test; NAFLD, non‐alcoholic fatty liver disease; NASH, non‐alcoholic steatohepatitis; NS, not significant.

Analysis of the 338 patients with NAFLD (exclusion of patients without steatosis) showed the same results as the entire cohort. No difference was found between women and men.

### *Results of other self‐reports*

**Figure 2:**
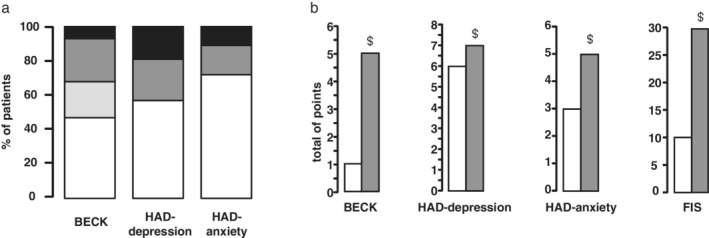
Evaluation of depression, anxiety, and fatigue through BECK, HAD, and FIS. (a) Distribution of BECK, HAD‐depression, and HAD‐anxiety values in severely obese patients. For BECK: no pathology = BECK <5; mild risk = BECK 5–7; moderate risk = BECK 8–15; severe risk = BECK >15. For HAD‐depression and HAD‐anxiety: no pathology = HAD <8; moderate risk = HAD 8–10; definite issue = HAD >10. (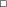), No pathology; (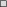), mild risk; (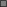), moderate risk; (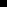), severe risk or definite issue. (b) Medians of BECK, HAD‐depression, HAD‐anxiety, and FIS in severely obese patients and in the control group. ^$^ *P* ≤ 0.00001 *versus* control group. (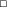), Control group; (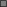), severely obese patients. BECK, Beck Depression Inventory; FIS, Fatigue Impact Scale; HAD, Hospital Anxiety and Depression Scale

According to BECK, 14 (8%) patients had “severe depression,” 48 (26%) had “moderate depression,” 37 (20%) had “mild depression,” and 84 (46%) had no depression (Fig. 2a). Median BECK was 5 [2–9] (Fig. 2b). According to the HAD‐depression scale, 37 (20%) patients had “definite depression,” 46 (25%) had “possible depression,” and 100 (55%) had normal test results (Fig. 2a). Median HAD‐depression was 7 [4–9] (Fig. 2b). Finally, the prevalence of significant depression was 34% for BECK and 20% for the HAD‐depression. Medians of BECK and HAD‐depression were similar in patients on an anxiolytic or sedative drug compared with patients not on an anxiolytic or sedative drug. In the control group, two subjects had “mild depression,” and all other subjects had negative pathological test results according to BECK (*P* < 0.00001 *vs* patients). According to the HAD‐depression, two subjects had “definite depression,” 3 had “possible depression,” and 24 had a normal test result (*P* < 0.00001 *vs* patients).

In obese patients, 21 (11.5%) had anxiety, 35 (19%) had “possible anxiety,” and 127 (69.5%) had a normal test (Fig. 2a). Median HAD‐anxiety was 5 [3–8]. The prevalence of significant anxiety was 28%. In the control group, no patient (*P* < 0.00001 *vs* patients) had a diagnosis of anxiety (Fig. 2b). The median of HAD‐anxiety was similar in patients on an anxiolytic or sedative drug compared with patients not on an anxiolytic or sedative drug.

The median FIS was 31 [14–70] for obese patients and 10 [3.5–23.5] (*P* < 0.00001 *vs* patients) for the control group (Fig. 2b). Patients treated with antidepressive drugs had significantly more fatigue (*P* = 0.02), but there was no influence of an anxiolytic or sedative drug.

No difference was found regardless of the presence of NAFLD; NASH; or fibrosis F0–F1 *versus* F2–F4, fibrosis F0–F2 *versus* F3–F4, presence of type 2 diabetes, or metabolic syndrome according to BECK or HAD or FIS (Table 3). Likewise, no correlation was found between grades of steatosis, stages of fibrosis, or NAS score. Results were similar between women and men. Finally, the use of antidepressive drugs or anxiolytics was not associated with the severity of NAFLD.

**Table 3:** Psychometric tests assessing depression, anxiety, and fatigue in 183 obese patients according to liver lesions, type 2 diabetes mellitus, or metabolic syndrome

	(*n*)	BECK	HAD‐depression	HAD‐anxiety	FIS
NAFLD	Yes (148)	5 [2–9]	7 [5–9]	5 [3–8]	31 [14–70]
	No (35)	5 [2–13]	7 [4–11]	5 [3–8]	320 [15–69]
NASH	Yes (26)	5.5 [2–8]	7 [5–10]	6 [4–8]	35.5 [14–73]
	No (157)	5 [2–9]	7 [4–9]	5 [3–8]	30.5 [13–70]
Fibrosis ≥ 2	Yes (37)	4 [2–8]	6 [4–8.5]	5 [3–8]	25 [14–73]
	No (146)	5 [2–9]	7 [5–9]	5 [3–8]	33 [14–69]
Type 2 diabetes	Yes (29)	4 [2–7]	6 [4–9.5]	5 [3.5–6.5]	27 [14–75.5]
	No (154)	5 [2–9]	7 [4–9]	5 [3–8]	33 [14–70]
Metabolic syndrome	Yes (89)	5 [2–9]	7 [5–10]	5 [3–8]	32.5 [15–72.5]
	No (94)	5 [2–10]	7 [4–9]	5 [3–8]	29 [10–62.5]

Data are expressed as medians with interquartile ranges and compared using the Mann–Whitney test. No difference was found for the psychometric tests between patients with and without NAFLD or NASH or fibrosis ≥2 or type 2 diabetes or metabolic syndrome.

BECK, Beck Depression Inventory; FIS, Fatigue Impact Scale; HAD, Hospital Anxiety and Depression Scale; NAFLD, non‐alcoholic fatty liver disease; NASH, non‐alcoholic steatohepatitis.

As expected, a positive correlation was found between the severity of BULIT and the severity of BECK (*r* = 0.5; *P* < 0.0001), HAD‐depression (*r* = 0.39; *P* < 0.0001), HAD‐anxiety (*r* = 0.38; *P* < 0.0001), and FIS (*r* = 0.35; *P* < 0.0001). Moreover, patients with more severe BED (BULIT ≥ 104) had more depression (*P* = 0.004 and 0.002 for BECK and HAD‐depression, respectively), more anxiety (*P* = 0.028), and more fatigue (*P* = 0.027) than patients with BULIT < 104. Similar results were found with different risks of BED (BULIT ≥ 88 or BULIT ≥ 73) (Table S2).

One year after bariatric surgery, among the exploratory follow‐up group of 15 patients, only 1 patient presented moderate risk of eating disorders, 1 patient had mild depression according to BECK, 1 patient had definite depression, and 2 had potential depression according to HAD. The patient with mild depression according to BECK was the same patient with definite depression according to the HAD. No patient had a diagnosis of anxiety.

## Discussion

Here, we report that the prevalence of BED (BULIT ≥ 73) was 38% in a well‐characterized cohort of 388 severely obese patients who had been referred for bariatric surgery.

Our cohort includes a majority of women, which is representative of European cohorts of morbidly obese patients.

Contrary to our hypothesis, the NAFLD features (presence of NAFLD *vs* no NAFLD, presence of NASH *vs* no NASH, fibrosis F2–F4 *vs* F0–F1, fibrosis F3–F4 *vs* F0–F2) of patients with BED were not more severe than patients without BED. A subgroup of 183 patients underwent further psychological assessment for depression, anxiety, and fatigue. Among these patients, 25–34% had significant depression, and 28% were anxious. A positive correlation between the severity of BED and the severity of depression, anxiety, and fatigue was found. We did not find any association between depression, anxiety, or fatigue and the liver pathology severity (presence of NAFLD *vs* no NAFLD, presence of NASH *vs* no NASH, fibrosis F2–F4 *vs* F0–F1, fibrosis F3–F4 *vs* F0–F2). All results were similar between women and men. Compared to healthy persons, severely obese patients had significantly more BED and were more depressed, more anxious, and more tired.

Only one pilot study had previously investigated BED and NAFLD. That study showed a higher prevalence of BED among 95 NAFLD patients than in the general population.^12^ However, the diagnosis of NAFLD was made inconsistently (28 patients with biopsy and 67 with imagery or noninvasive test).^12^ In contrast, all 388 patients in our cohort had a biopsy. Unlike our initial hypothesis, we did not show a correlation between BED and severity of NAFLD or the presence of metabolic syndrome or the presence of type 2 diabetes. In contrast with our study, Hudson *et al*. showed that BED could confer a risk of components of the metabolic syndrome in 268 patients with a clinical diagnosis of BED.^13^ The difference in our results could be because the assessment of BED was made during an interview by a psychiatrist using Diagnostic and Statistical Manual of Mental Disorders (DSM)‐IV. Moreover, patients in the study of Hudson *et al*. were more severe than those in our study because we excluded patients with symptomatic BED.

We found a correlation between the severity of BED and the severity of depression, anxiety, and fatigue as previously published in several studies on overweight or obese patients.^13^, ^30^, ^31^, ^32^, ^33^ However, the association between NAFLD and depression, anxiety, or fatigue has rarely been studied.^34^, ^35^, ^36^ The evaluation of psychological disorders had two complementary interests: to improve the knowledge of the association between psychological disorders and NAFLD and also to ensure that the anxiodepressive participation was not the explanatory parameter linking NAFLD and BED. Depression, anxiety, fatigue, and BED could also have had a synergistic association in the presence of NASH and fibrosis in severely obese subjects.

A recent meta‐analysis estimated that the prevalence of depression was 19% among patients undergoing bariatric surgery.^37^ These results are comparable to ours. Patients with NAFLD seemed to be more depressed and more anxious than the general population. A study with 36 NASH and 36 matched controls showed more severe histological features in patients with major depressive disorder and generalized anxiety disorder determined by a structured interview. More than 50% of NASH patients had major depressive disorder, which was very high.^35^ Youssef *et al*. showed, in 567 biopsy‐proven NAFLD patients, that depression or anxiety was not associated with the severity of NAFLD, the presence of NASH, or sinusoidal fibrosis. In contrast to our study, they showed an association between depression with ballooning hepatocytes and portal fibrosis. They used the same screening test as we did (HAD). The differences between their results and our own could be explained because their cohort differed from ours as they included morbidly obese patients (mean BMI 48.3 ± 7.7 kg/m^2^) with suspected NAFLD and also obese patients (mean BMI 35.6 ± 7.6 kg/m^2^) who had a clinical indication for percutaneous liver biopsy as determined by one of the attending hepatologists. No information was given about the number of patients in each group. Possibly due to this, their patients were more severely affected than ours: 14% *versus* 3.9% of F3‐F4 fibrosis and 36% *versus* 16% with a type 2 diabetes, respectively. Very interestingly, in the subgroup analysis of morbidly obese patients, no significant association was found with liver damage, which is similar to our study.^36^ Fatigue is a very common sensation, but this is the first time, to our knowledge, that fatigue has been studied in severely obese patients with liver biopsies. Newton *et al*. showed that fatigue, using FIS, was increased in 156 biopsy‐proven NAFLD patients with a mean BMI of 32 ± 6 kg/m^2^ compared to healthy subjects. Consistent with our study, they showed that FIS was not associated with the severity of histological features (steatosis, fibrosis, or steatohepatitis).^34^ The lack of significant differences between patients with or without NAFLD in our cohort could be explained by the fact that our cohort is mainly composed of morbidly obese patients, and obesity per se could be associated with fatigue. These patients have a very high amount of adipose tissue, and unhealthy obesity is a pathologic state characterized by metabolic inflammation,^38^ and this inflammation of adipose tissue is associated with a release of cytokines (tumor necrosis factor, interleukin, osteopontin, etc.).^39^ These adipokines activate key signaling molecules in both metabolic and inflammatory pathways (NF‐κB) and acute phase proteins.^40^ Among these latter molecules, we have previously shown that the C‐reactive protein was associated with the severity of obesity and steatosis but not with the presence of NASH.^39^, ^41^ Similarly, psychological abnormalities may be more closely associated with obesity than with liver features. A comparison group of patients with NAFLD but without severe obesity could have been interesting.

Some limitations in our study must be noted. First, patients with clinical bulimia nervosa were contraindicated for bariatric surgery and were not included in our study. A control positive group of severely obese bulimic patients may have been an interesting comparison. Secondly, the BULIT was validated to diagnose eating disorders according to DSM‐III. The evolution of the DSM (now DSM‐V) and the new subgroup of eating disorders had not been evaluated. Thirdly, our study may have inherent sampling and reporting biases in that only those patients who completed self‐reported questionnaire data were included. Fourthly, although no information about education level and job status was obtained from patients, previous studies on severely obese patients showed no impact of these factor on the occurrence of BED.^42^

Our study has several strengths; first, it included 388 patients with a systematic liver biopsy, reviewed by a single pathologist, while the other studies did not have biopsy or were only on a small number of patients. Second, we used psychological tests that have been validated in the literature. BULIT, Beck, and HAD have been used previously in obese populations and are validated for use in severely obese populations.^21^, ^24^, ^43^ Third, we had a control group matched for the age and gender.

In conclusion, contrary to our hypothesis, mild BED appeared to be more closely associated with obesity than with liver pathology in severely obese patients. However, more studies could be interesting on patients with symptomatic BED and with nonsevere obese NAFLD.

## Supporting information


**Appendix** **S1** Supplementary methods.

**Table S1** Comparison of an exploratory follow‐up cohort of 15 patients *versus* the patients at baseline.
**Table S2** Comparison of BECK, HAD‐Depression, HAD‐Anxiety, and FIS according to the severity of BULIT.
