# Supplementary material for: No association between binge eating disorder and severity of non‐alcoholic fatty liver disease in severely obese patients
Source: JGH Open. 2020 Mar 1;4(3):525–31. doi: 10.1002/jgh3.12309 (PMC7273712; doi:10.1002/jgh3.12309)
Supplement: Supplementary file 1 — Appendix S1 Supplementary methods. [file JGH3-4-525-s001.docx]

**No association between binge eating disorder and severity of Nonalcoholic Fatty Liver Disease in severely obese patients**

CANIVET, Clémence M (1); PERNEY, Pascal (2,3); CHERICK, Faredj (4); ORLOWSKI, Magalie (4); PATOURAUX, Stéphanie (1); BAILLY-MAITRE, Béatrice (5); TRAN, Albert (1); IANNELLI, Antonio (1); GUAL, Philippe $ (5); ANTY, Rodolphe $ (1)

Supplementary methods:

All patients met the 1991 NIH Consensus Conference guidelines for gastrointestinal surgery for obesity.^1^ All patients were negative for hepatitis B and C viral markers, for auto-antibodies indicative of autoimmune hepatitis and had negligible alcohol consumption (< 20 g/day in women and < 30g/day in men). Alcohol abuse was also excluded by interviewing the patients’ relatives. Patients with a history of inflammatory disease (including rheumatoid arthritis, systemic lupus erythematous, and inflammatory bowel disease), current infections, recent history of cancer (< 5 years), and severe pulmonary or cardiac disease were not enrolled in the study. All patients underwent bariatric surgery and a surgical liver biopsy was obtained during the operation. Written informed consent was obtained from all patients. Metabolic syndrome was defined as the presence of at least three of the following parameters: elevated waist circumference (≥ 94 cm in men or ≥ 80 cm in women), elevated blood pressure (systolic blood pressure ≥ 130 mmHg or diastolic blood pressure ≥ 85 mmHg or antihypertensive drug), elevated blood glucose (≥ 100 mg/dL or anti-diabetic drug), elevated triglycerides (≥ 150 mg/dl or lipid-lowering drug), and low HDL cholesterol (< 40 mg/dL in men or < 50 mg/dL in women or lipid-lowering drug).^2^ Noteworthy, all the obese patients from the cohort had a waist circumference above the threshold of the metabolic syndrome definition. Type 2 diabetes was defined by a fasting blood sugar level ≥ 126 mg/dL.

*Pathological liver assessment*: hepatic wedges were at least 10 mm long. The diagnosis was retained based on the SAF score for diagnosis of NASH in morbidly obese patients. This algorithm includes steatosis (0: < 5%; 1: 5-33%; 2: 34-66% and 3: 67-100%), activity with ballooning (0: absence; 1: hepatocytes with a rounded shape and a pale cytoplasm with a size similar to that of normal hepatocytes; 2: same as 1 but enlarged hepatocytes at least 2-fold that of normal cells) and inflammation (0: no inflammation; 1: less than 2 foci per 20x; 2: at least 2 foci per 20x) and fibrosis. NAFLD was defined as at least steatosis grade 1. NASH was defined as at least steatosis grade 1, hepatocellular ballooning grade 1 and lobular inflammation grade 1.^3^ The NASH Activity Score was also determined as previously described.^4^ Liver fibrosis was assessed with Sirius red staining and was classified into seven stages according to the NASH Clinical Research Network Scoring System Definition and Scores as follows: F0: no fibrosis, F1a: mild sinusoidal fibrosis, F1b: moderate sinusoidal fibrosis, F1c: peri-portal sinusoidal fibrosis, F2: sinusoidal fibrosis and peri-portal sinusoidal fibrosis, F3: bridging fibrosis, F4: cirrhosis. In this study, “significant” fibrosis was considered if fibrosis was ≥ F2.

References:

1. NIH conference. Gastrointestinal surgery for severe obesity. Consensus Development Conference Panel. Ann Intern Med. 1991 Dec 15;115(12):956–61.

2. Alberti KGMM, Eckel RH, Grundy SM, Zimmet PZ, Cleeman JI, Donato KA, et al. Harmonizing the metabolic syndrome: a joint interim statement of the International Diabetes Federation Task Force on Epidemiology and Prevention; National Heart, Lung, and Blood Institute; American Heart Association; World Heart Federation; International Atherosclerosis Society; and International Association for the Study of Obesity. Circulation. 2009 Oct 20;120(16):1640–5.

3. Bedossa P, Poitou C, Veyrie N, Bouillot J-L, Basdevant A, Paradis V, et al. Histopathological algorithm and scoring system for evaluation of liver lesions in morbidly obese patients. Hepatology. 2012 Nov;56(5):1751–9.

4. Kleiner DE, Brunt EM, Van Natta M, Behling C, Contos MJ, Cummings OW, et al. Design and validation of a histological scoring system for nonalcoholic fatty liver disease. Hepatology. 2005 Jun;41(6):1313–21.
