# Supplementary material for: No association between binge eating disorder and severity of non‐alcoholic fatty liver disease in severely obese patients
Source: JGH Open. 2020 Mar 1;4(3):525–31. doi: 10.1002/jgh3.12309 (PMC7273712; doi:10.1002/jgh3.12309)
Supplement: Supplementary file 2 — Table S1 Comparison of an exploratory follow‐up cohort of 15 patients versus the patients at baseline. Table S2 Comparison of BECK, HAD‐Depression, HAD‐Anxiety, and FIS according to the severity of BULIT. [file JGH3-4-525-s002.docx]

Supplementary table 1: Comparison of an exploratory follow-up cohort of 15 patients versus the patients at baseline

| Variables | Baseline | Month 12 | p |
| --- | --- | --- | --- |
| Total number of subjects | 389 | 15 | - |
| Female (%) | 315 (81) | 11 (73) | NS |
| Age at baseline (years) | 40 [38-41] | 48 [43-61] | 0.0043 |
| BMI at baseline (kg/m2) | 41.8 [41.2-42.3] | 41.5 [37.8-48.5] | NS |
| BMI at time of comparison (kg/m2) | 41.8 [41.2-42.3] | 27.1 [25.5-32.0] | <0.000001 |

Median with interquartile ranges

Supplementary Table 2: Comparison of BECK, HAD-Depression, HAD-Anxiety and FIS according to the severity of BULIT.

|  | BECK | HAD-Depression | HAD-Anxiety | FIS |
| --- | --- | --- | --- | --- |
| BULIT ≥104 | 13  [6-18] | 10.5  [8.5-15] | 9.5  [5-15.5] | 74  [45-81] |
| BULIT < 104 | 5  [2-9] | 7  [4-9] | 5  [3-8] | 30  [14-69] |
| *p* | 0.004 | 0.002 | 0.028 | 0.027 |
|  |  |  |  |  |
| BULIT ≥ 88 | 11.5  [7-16] | 9.5  [8-12] | 7  [5-10] | 60.5  [38-81] |
| BULIT < 88 | 4  [2-8] | 7  [4-9] | 5  [3-8] | 25.5  [12-60] |
| *p* | < 0.00001 | 0.00004 | 0.0003 | 0.0003 |
|  |  |  |  |  |
| BULIT ≥ 73 | 9  [5-13] | 8.5  [7-11] | 7  [5-10] | 58  [26-82.5] |
| BULIT < 73 | 3  [1-6] | 6  [4-8] | 4  [3-7] | 22  [10-51] |
| *p* | < 0.00001 | 0.00003 | 0.00001 | < 0.00001 |

BULIT ≥ 104 = probable diagnosis of bulimia; BULIT ≥ 88 = high risk of binge eating disorder; BULIT ≥ 73 = moderate risk of binge eating disorder. Data are expressed as median with interquartile ranges and compared using the Mann Whitney test for quantitative values.
